# Crystal structure of (1*S*,2*S*,2′*R*,3a′*S*,5*R*)-2′-[(5-bromo-1*H*-indol-3-yl)meth­yl]-2-isopropyl-5,5′-dimethyl­dihydro-2′*H*-spiro­[cyclo­hexane-1,6′-imidazo[1,5-*b*]isoxazol]-4′(5′*H*)-one

**DOI:** 10.1107/S2056989016010872

**Published:** 2016-07-12

**Authors:** Siwar Ghannay, Jihed Brahmi, Soumaya Nasri, Kaïss Aouadi, Erwann Jeanneau, Moncef Msaddek

**Affiliations:** a Université de Monastir, Laboratoire de Synthése Hétérocyclique, Produits Naturels et Réactivités, Faculté des Sciences de Monastir, Avenue de l’Environnement, 5000 Monastir, Tunisia; b Laboratoire de Physico-chimie des Matériaux, Faculté des Sciences de Monastir, Avenue de l’Environnement, 5019 Monastir, University of Monastir, Tunisia; c Université Lyon 1, Centre de Diffractométrie Henri Longchambon, Bâtiment 305, 43 boulevard du 11 Novembre 1918, F-69622 Villeurbanne Cedex, France

**Keywords:** crystal structure, isoxazolidines, 1,3-dipolar cyclo­addition, chiral nitrone, hydrogen bonding

## Abstract

In the title compound, C_24_H_32_BrN_3_O_2_, the six-membered cyclo­hexane ring adopts a chair conformation and the isoxasolidine ring adopts a twisted conformation. The mol­ecule has five chiral centres and the absolute configuration has been determined in this analysis. The mol­ecular structure is stabilized by weak intra­molecular C—H⋯O and C—H⋯N contacts. In the crystal, mol­ecules are linked by N—H⋯N and C—H⋯O hydrogen bonds, forming undulating sheets parallel to the *bc* plane.

## Abstract 2

The absolute structure for the title compound, which has five chiral centres has been determined in this analysis. The supra­molecular architecture comprises parallel zigzag chains formed through N—H⋯N and C—H⋯O hydrogen bonds, as well as intra­molecular C—H⋯O, C—H⋯N and C—H⋯π inter­actions.

## Chemical context   

1,3-Dipolar cyclo­additions of alkenes with nitro­nes produce substituted isoxazolidines. Nitrone cyclo­adducts offer a general route to natural and unnatural amino acids (Aouadi *et al.*, 2006 ▸, 2007 ▸) through opening of the isoxazolidine ring, usually by reductive cleavage of the weak N—O bond. Consequently, isoxazolidines have been used as key inter­mediates for the synthesis of various natural products, anti­fungals (Kumar *et al.*, 2003 ▸), anti-tuberculosis (Kumar *et al.*, 2010 ▸) and anti­viral agents (Loh *et al.*, 2010 ▸). We present herein the synthesis, the mol­ecular structure and the spectroscopic data of the title compound, C_24_H_32_BrN_3_O_2_, (I).
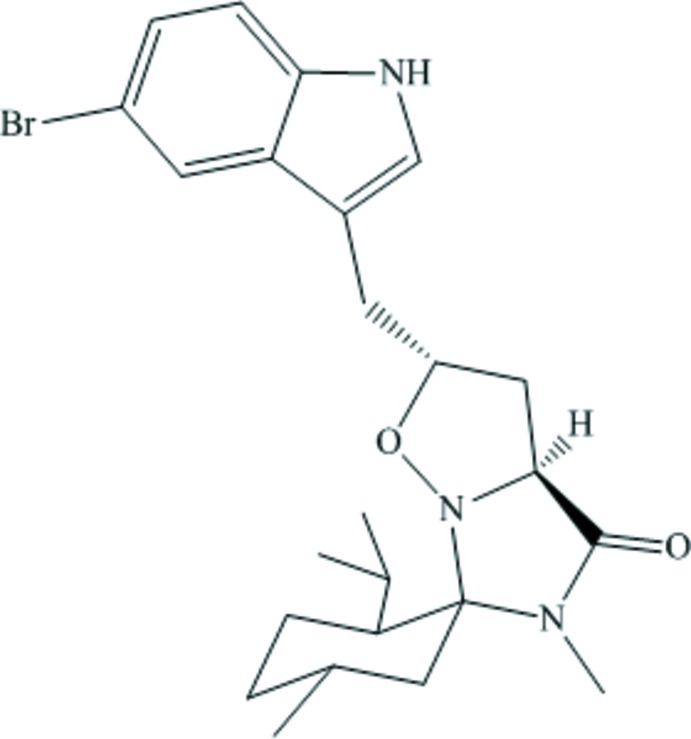


## Structural commentary   

**Figure 1:**
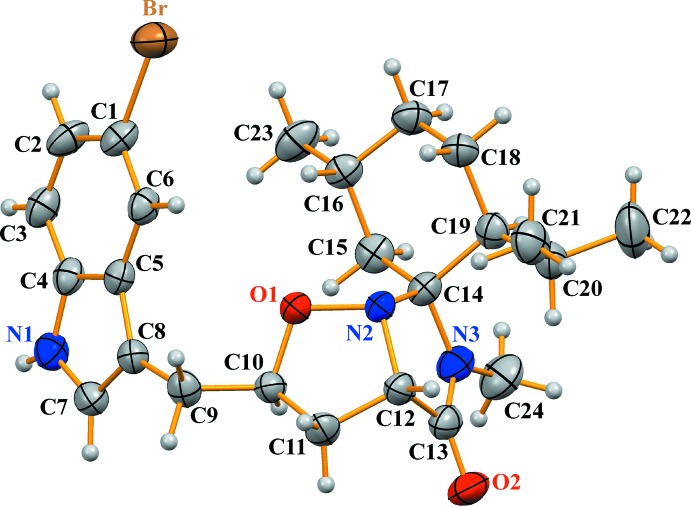
The mol­ecular structure of the title compound, showing the atom labelling. Displacement ellipsoids are drawn at the 40% probability level.

In the title compound (I) (Fig. 1 ▸), the five-membered isoxazolidine ring has a twist conformation. The O1—N2 bond length in the isoxazolidine ring is 1.475 (6) Å which is close to the values in related compounds (Lee *et al.*, 2010 ▸; Molander & Cavalcanti, 2013 ▸). The cyclo­hexane ring adopts a chair conformation. The dihedral angle between the mean planes of the isoxazolidine and imidazolidinone rings is 73.1 (3)° while the C8—C9—C10—O1 torsion angle is 74.7 (7)°. In the molecule there are some short C—H⋯O and C—H⋯N contacts present (Table 1 ▸). The absolute configuration of (I) has been confirmed as C10(*R*),C12(*S*),C14(*S*),C16(*R*),C19(*S*) for the five arbitrarily numbered chiral centres in the mol­ecule.

## Supra­molecular features   

**Figure 2:**
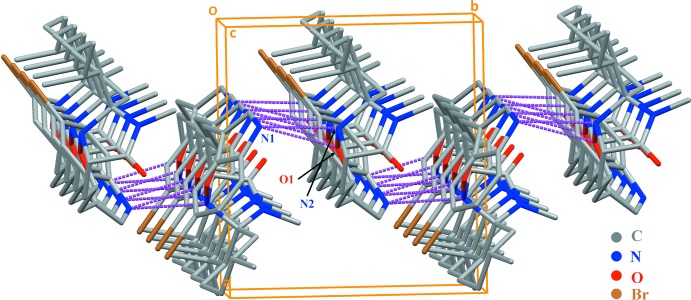
A view of the title structure, showing the mol­ecules of the title compound arranged in zigzag parallel chains sustained by weak N—H⋯N and N—H⋯O hydrogen bonds.

In the crystal packing of (I), the mol­ecules are linked through an inter­molecular N1—H*N*1⋯N2^i^ hydrogen bond (Table 1 ▸) and a weak N1—H*N*1⋯O1^i^ inter­action [3.053 (8) Å], forming undulating sheets parallel to the *bc* plane (Fig. 2 ▸). Within the chains, the mol­ecules are stabilized by a weak inter­molecular C3—H3⋯O2^ii^ hydrogen bond (Table 1 ▸). Also present in the crystal are 39.3 Å^3^ solvent-accessible voids.

## Synthesis and crystallization   

**Figure 3:**
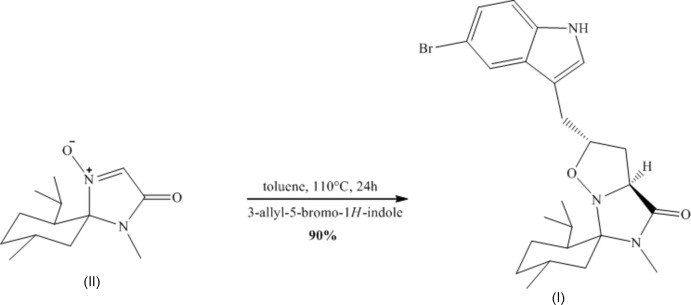
Reaction scheme for the synthesis of compound (I).

To a solution of 3-allyl-5-bromo-1*H*-indole (1.40 mmol, 330 mg) in toluene (10 mL) was added 5(*R*),6(*S*),9(*R*)-6-isopropyl-1,9-dimethyl-1,4-diazo­aspiro­[4,5]-decan-1-ene-3-one-1-oxide (II) (1.19 mmol, 285 mg) and the mixture was stirred and heated at reflux at 383 K for 24 h under argon. TLC indicated the complete conversion of (II). The solution obtained was concentrated and the residue was purified by flash chromatography (petroleum ether–ethyl acetate 7:3) to afford the cyclo­adduct (I) as a white solid (507 mg, 90% yield) (Fig. 3 ▸). Colorless plate-shaped crystals of (I) were obtained by slow evaporation of a diethyl ether solution.

## Spectroscopic investigations   

NMR spectra were recorded on a Bruker Avance II 300 MHz spectrometer operating at 300 MHz for ^1^H and 75.46 MHz for ^13^C and were referenced to tetra­methyl­silane (δ = 0 p.p.m.). High-resolution (HR–ESI–QToF) mass spectra were recorded using a Bruker Micro ToF-Q II XL spectrometer.

The^1^H NMR spectrum of (I) shows the presence of an NH proton at 8.32 p.p.m. and the^13^C NMR spectrum confirms the existence of the C3 and C5 stereogenic centres at 66.4 p.p.m. and 78.0 p.p.m., respectively. The spectroscopic measurements are consistent with the crystal structure of (I). High-resolution mass spectrometry in the positive-ion mode exhibits an [*M*+H]^+^ fragment of 474.1759 *m*/*z* which is very close to the calculated value of 474.1756 *m*/*z*.

*R*_f_ = 0.33 (PE–EtOAc 7:3). NMR ^1^H (300 MHz, CDCl_3_) *δ(*p.p.m.): 0.62 (*d*, 3H, *J* = 6.6 Hz), 0.83 (*d*, 3H, *J* = 6.6 Hz), 0.85 (*m*, 1H), 0.86 (*d*, 3H, *J* = 6.3 Hz), 1.11 (*t*, 1H, *J* = 12.3 Hz), 1.21–1.43 (*m*, 2H), 1.57–1.67 (*m*, 1H), 1.70–1.83 (*m*, 3H), 1.90–2.02 (*m*, 1H), 2.26 (*ddd*, 1H, *J* = 8.7 Hz, 10.2 Hz and 12 Hz), 2.69 (*s*, 3H, NCH_3_), 2.67–2.72 (*m*, 1H), 2.93–2.97 (*m*, 2H), 3.88–3.97 (*m*, 1H), 4.01 (*brd*, 1H, *J* = 8.4 Hz), 7.02 (*brd*, 1H, *J* = 4.8 Hz), 7.21 (*m*, 2H), 7.74 (*brd*, 1H, *J* = 1.8 Hz), 8.32 (*brs*, 1H, NH). ^13^C NMR (CDCl_3_, 75.46 MHz) *δ(*p.p.m.): 18.3, 22.0, 22.2 (CH_2_), 24.1, 24.3, 26.0, 28.1, 29.6, 34.5 (CH_2_), 38.8 (CH_2_), 40.3 (CH_2_), 48.0, 66.4, 78.0, 90.0, 112.3, 112.4, 112.7, 121.6, 123.5, 124.7, 129.1, 134.6, 173.0 (C=O). [α] = + 43.7 (*c =* 1, CH_2_Cl_2_).

## Refinement   

Crystal data, data collection and structure refinement details are summarized in Table 2 ▸. All hydrogen atoms attached to C atoms were fixed geometrically and treated as riding with C—H = 0.98 Å (methine), 0.97 Å (methyl­ene), 0.96 Å (meth­yl) and 0.93 Å (aromatic), with *U*_iso_(H) = 1.2*U*_eq_(C)(methine, methyl­ene, aromatic) or 1.5*U*_eq_C(meth­yl). The H atom on the nitro­gen N1 of the indole ring was found in a difference-Fourier map but was subsequently refined with the coordinates and isotropic displacement parameter also riding with *U*_iso_ = 1.2 *U*_eq_(N). The bond length N1—H*N*1 was restrained to ensure proper geometry using the DFIX instruction of *SHELXL2014/7* (Sheldrick, 2015 ▸). The absolute structure Flack parameter [−0.013 (13) for 1005 quotients (Parsons *et al.*, 2013 ▸)] confirmed the configuration of the mol­ecule as C10(*R*),C12(*S*),C14(*S*),C16(*R*),C19(*S*) for the five arbitrarily numbered chiral centres in the mol­ecule.

## Supplementary Material

Crystal structure: contains datablock(s) I. DOI: 10.1107/S2056989016010872/zs2365sup1.cif

Structure factors: contains datablock(s) I. DOI: 10.1107/S2056989016010872/zs2365Isup2.hkl

Supporting information file. DOI: 10.1107/S2056989016010872/zs2365Isup3.cml

CCDC reference: 1490701

Additional supporting information: crystallographic information; 3D view; checkCIF report

## Figures and Tables

**Table 1:** Hydrogen-bond geometry (Å, °)

*D*—H⋯*A*	*D*—H	H⋯*A*	*D*⋯*A*	*D*—H⋯*A*
N1—H*N*1⋯N2^i^	0.89	2.34	3.087 (8)	141
C3—H3⋯O2^ii^	0.93	2.42	3.292 (9)	156
C16—H16⋯O1	0.98	2.55	3.091 (8)	115
C20—H20⋯N3	0.98	2.54	3.032 (10)	111
C21—H21*A*⋯N2	0.96	2.60	3.236 (9)	124

Symmetry codes: (i) 
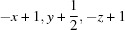
; (ii) 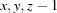.

**Table 2:** Experimental details

Crystal data	
Chemical formula	C_24_H_32_BrN_3_O_2_
*M*_r_	474.43
Crystal system, space group	Monoclinic, *P*2_1_
Temperature (K)	293
*a*, *b*, *c* (Å)	10.2640 (5), 9.6480 (5), 12.0480 (5)
β (°)	96.204 (5)
*V* (Å^3^)	1186.09 (10)
*Z*	2
Radiation type	Mo *K*α
μ (mm^−1^)	1.76
Crystal size (mm)	0.46 × 0.39 × 0.11

Data collection	
Diffractometer	Oxford Diffraction Xcalibur Atlas Gemini Ultra CCD
Absorption correction	Multi-scan (*SCALEPACK*; Otwinowski *et al.*, 1997 ▸)
*T*_min_, *T*_max_	0.455, 0.802
No. of measured, independent and observed [*I* > 2σ(*I*)] reflections	10653, 4337, 2924
*R*_int_	0.104
(sin θ/λ)_max_ (Å^−1^)	0.617

Refinement	
*R*[*F* ^2^ > 2σ(*F* ^2^)], *wR*(*F* ^2^), *S*	0.053, 0.135, 0.97
No. of reflections	4337
No. of parameters	272
No. of restraints	2
H-atom treatment	H-atom parameters constrained
Δρ_max_, Δρ_min_ (e Å^−3^)	0.31, −0.51
Absolute structure	Flack *x* determined using 1005 quotients [(*I* ^+^)−(*I* ^−^)]/[(*I* ^+^)+(*I* ^−^)] (Parsons *et al.*, 2013 ▸)
Absolute structure parameter	−0.013 (13)

Computer programs: *CrysAlis PRO* (Agilent, 2013 ▸), *SIR2011* (Burla *et al.*, 2012 ▸), *SHELXL2014* (Sheldrick, 2015 ▸), *ORTEPIII* (Burnett & Johnson, 1996 ▸) and *ORTEP-3 for Windows* and *WinGX* (Farrugia, 2012 ▸).

## supplementary crystallographic information

### Crystal data

**Table:**

C_24_H_32_BrN_3_O_2_	*F*(000) = 496
*M**_r_* = 474.43	*D*_x_ = 1.328 Mg m^−^^3^
Monoclinic, *P*2_1_	Mo *K*α radiation, λ = 0.71073 Å
*a* = 10.2640 (5) Å	Cell parameters from 8154 reflections
*b* = 9.6480 (5) Å	θ = 1.0–27.9°
*c* = 12.0480 (5) Å	µ = 1.76 mm^−^^1^
β = 96.204 (5)°	*T* = 293 K
*V* = 1186.09 (10) Å^3^	Plate, colorless
*Z* = 2	0.46 × 0.39 × 0.11 mm

### Data collection

**Table:**

Oxford Diffraction Xcalibur Atlas Gemini Ultra CCD diffractometer	4337 independent reflections
Radiation source: Enhance (Mo) X-ray source)	2924 reflections with *I* > 2σ(*I*)
Graphite monochromator	*R*_int_ = 0.104
ω/2θ scans	θ_max_ = 26.0°, θ_min_ = 2.7°
Absorption correction: multi-scan (SCALEPACK; Otwinowski *et al.*, 1997)	*h* = −12→12
*T*_min_ = 0.455, *T*_max_ = 0.802	*k* = −11→11
10653 measured reflections	*l* = −13→14

### Refinement

**Table:**

Refinement on *F*^2^	Hydrogen site location: mixed
Least-squares matrix: full	H-atom parameters constrained
*R*[*F*^2^ > 2σ(*F*^2^)] = 0.053	*w* = 1/[σ^2^(*F*_o_^2^) + (0.0631*P*)^2^] where *P* = (*F*_o_^2^ + 2*F*_c_^2^)/3
*wR*(*F*^2^) = 0.135	(Δ/σ)_max_ < 0.001
*S* = 0.97	Δρ_max_ = 0.31 e Å^−^^3^
4337 reflections	Δρ_min_ = −0.51 e Å^−^^3^
272 parameters	Absolute structure: Flack *x* determined using 1005 quotients [(*I*^+^)-(*I*^-^)]/[(*I*^+^)+(*I*^-^)] (Parsons *et al.*, 2013)
2 restraints	Absolute structure parameter: −0.013 (13)

### Special details

**Table:**

Geometry. All esds (except the esd in the dihedral angle between two l.s. planes) are estimated using the full covariance matrix. The cell esds are taken into account individually in the estimation of esds in distances, angles and torsion angles; correlations between esds in cell parameters are only used when they are defined by crystal symmetry. An approximate (isotropic) treatment of cell esds is used for estimating esds involving l.s. planes.

### Fractional atomic coordinates and isotropic or equivalent isotropic displacement parameters (Å^2^)

**Table:**

	*x*	*y*	*z*	*U*_iso_*/*U*_eq_	
Br	0.17985 (7)	0.23216 (8)	0.27079 (6)	0.0716 (3)	
O1	0.4307 (4)	0.4046 (5)	0.6482 (4)	0.0455 (11)	
N3	0.3297 (6)	0.6276 (6)	0.8208 (5)	0.0559 (16)	
O2	0.5063 (6)	0.6393 (6)	0.9554 (4)	0.0728 (16)	
N2	0.3578 (5)	0.4065 (6)	0.7471 (4)	0.0419 (12)	
N1	0.6516 (6)	0.6022 (6)	0.3324 (5)	0.0521 (15)	
C14	0.2644 (7)	0.5267 (7)	0.7427 (5)	0.0447 (15)	
C6	0.4147 (7)	0.3475 (8)	0.3841 (6)	0.0472 (16)	
H6	0.4007	0.2871	0.4418	0.057*	
C9	0.6468 (6)	0.4016 (8)	0.5952 (5)	0.0478 (16)	
H9A	0.7375	0.4153	0.6248	0.057*	
H9B	0.6317	0.3025	0.5885	0.057*	
C1	0.3301 (7)	0.3486 (8)	0.2869 (6)	0.0523 (18)	
C11	0.5918 (7)	0.4126 (9)	0.7977 (6)	0.0513 (18)	
H11A	0.6196	0.3164	0.8013	0.062*	
H11B	0.6594	0.4698	0.8372	0.062*	
C10	0.5591 (6)	0.4605 (7)	0.6774 (5)	0.0426 (15)	
H10	0.5572	0.5619	0.6734	0.051*	
C8	0.6248 (6)	0.4659 (7)	0.4806 (5)	0.0439 (15)	
C13	0.4388 (7)	0.5769 (8)	0.8831 (6)	0.0523 (18)	
C19	0.1319 (7)	0.4750 (8)	0.7834 (5)	0.0485 (17)	
H19	0.0774	0.5582	0.7854	0.058*	
C5	0.5213 (6)	0.4378 (7)	0.3948 (5)	0.0428 (15)	
C12	0.4609 (6)	0.4315 (8)	0.8439 (5)	0.0471 (16)	
H12	0.4518	0.3648	0.9039	0.057*	
C16	0.1585 (6)	0.4977 (7)	0.5389 (5)	0.0472 (16)	
H16	0.2112	0.4153	0.5275	0.057*	
C15	0.2358 (7)	0.5914 (7)	0.6262 (6)	0.0500 (17)	
H15A	0.3184	0.6159	0.5992	0.060*	
H15B	0.1867	0.6764	0.6328	0.060*	
C7	0.6991 (6)	0.5668 (8)	0.4386 (6)	0.0503 (17)	
H7	0.7730	0.6064	0.4776	0.060*	
C4	0.5407 (7)	0.5268 (7)	0.3039 (6)	0.0459 (16)	
C18	0.0585 (7)	0.3816 (8)	0.6964 (6)	0.0544 (18)	
H18A	−0.0241	0.3541	0.7220	0.065*	
H18B	0.1096	0.2983	0.6884	0.065*	
C3	0.4561 (8)	0.5248 (8)	0.2046 (6)	0.056 (2)	
H3	0.4705	0.5828	0.1455	0.068*	
C17	0.0316 (6)	0.4521 (9)	0.5829 (6)	0.0574 (19)	
H17A	−0.0146	0.3883	0.5302	0.069*	
H17B	−0.0241	0.5322	0.5896	0.069*	
C21	0.1702 (8)	0.2603 (9)	0.9120 (6)	0.071 (2)	
H21A	0.2474	0.2388	0.8770	0.107*	
H21B	0.1833	0.2340	0.9892	0.107*	
H21C	0.0968	0.2105	0.8754	0.107*	
C2	0.3522 (8)	0.4357 (8)	0.1965 (6)	0.061 (2)	
H2	0.2954	0.4324	0.1309	0.073*	
C20	0.1432 (7)	0.4168 (9)	0.9030 (6)	0.0554 (18)	
H20	0.2168	0.4641	0.9457	0.066*	
C23	0.1317 (8)	0.5741 (9)	0.4278 (6)	0.068 (2)	
H23A	0.2133	0.6015	0.4022	0.102*	
H23B	0.0854	0.5139	0.3736	0.102*	
H23C	0.0796	0.6549	0.4376	0.102*	
C22	0.0205 (10)	0.4502 (13)	0.9589 (8)	0.097 (3)	
H22A	0.0032	0.5479	0.9535	0.145*	
H22B	−0.0527	0.4001	0.9222	0.145*	
H22C	0.0338	0.4236	1.0361	0.145*	
C24	0.2777 (9)	0.7638 (8)	0.8427 (8)	0.081 (3)	
H24A	0.2015	0.7815	0.7912	0.121*	
H24B	0.2542	0.7668	0.9177	0.121*	
H24C	0.3431	0.8329	0.8338	0.121*	
HN1	0.6758	0.6699	0.2885	0.07 (2)*	

### Atomic displacement parameters (Å^2^)

**Table:**

	*U*^11^	*U*^22^	*U*^33^	*U*^12^	*U*^13^	*U*^23^
Br	0.0695 (5)	0.0753 (5)	0.0667 (5)	−0.0179 (5)	−0.0081 (3)	0.0009 (5)
O1	0.042 (3)	0.058 (3)	0.036 (3)	−0.003 (2)	0.0033 (19)	0.000 (2)
N3	0.066 (4)	0.049 (4)	0.052 (4)	−0.006 (3)	0.004 (3)	−0.008 (3)
O2	0.078 (4)	0.092 (4)	0.047 (3)	−0.029 (3)	−0.001 (3)	−0.015 (3)
N2	0.042 (3)	0.053 (3)	0.031 (3)	−0.002 (3)	0.002 (2)	0.001 (2)
N1	0.059 (4)	0.049 (4)	0.049 (4)	−0.007 (3)	0.012 (3)	0.004 (3)
C14	0.051 (4)	0.046 (4)	0.037 (4)	0.000 (3)	0.002 (3)	−0.003 (3)
C6	0.058 (4)	0.048 (4)	0.037 (4)	−0.004 (3)	0.009 (3)	0.005 (3)
C9	0.043 (4)	0.056 (4)	0.045 (4)	0.012 (3)	0.006 (3)	0.011 (3)
C1	0.067 (5)	0.047 (4)	0.042 (4)	−0.001 (4)	−0.001 (3)	−0.007 (3)
C11	0.045 (4)	0.070 (5)	0.037 (4)	0.003 (4)	−0.002 (3)	0.007 (4)
C10	0.040 (3)	0.049 (4)	0.038 (4)	−0.006 (3)	0.003 (3)	0.000 (3)
C8	0.042 (3)	0.049 (4)	0.041 (4)	0.000 (3)	0.009 (3)	−0.001 (3)
C13	0.058 (4)	0.062 (5)	0.038 (4)	−0.013 (4)	0.008 (3)	−0.001 (3)
C19	0.048 (4)	0.052 (4)	0.046 (4)	0.009 (3)	0.006 (3)	0.002 (3)
C5	0.048 (4)	0.043 (4)	0.038 (4)	0.004 (3)	0.008 (3)	−0.003 (3)
C12	0.046 (4)	0.060 (5)	0.034 (3)	−0.009 (3)	0.003 (3)	0.003 (3)
C16	0.051 (4)	0.044 (4)	0.045 (4)	0.006 (3)	−0.003 (3)	0.004 (3)
C15	0.058 (4)	0.037 (4)	0.054 (5)	0.008 (3)	0.003 (3)	0.008 (3)
C7	0.047 (4)	0.054 (4)	0.051 (4)	−0.005 (4)	0.009 (3)	−0.004 (3)
C4	0.057 (4)	0.038 (4)	0.043 (4)	0.001 (3)	0.011 (3)	−0.003 (3)
C18	0.044 (4)	0.067 (5)	0.050 (4)	−0.002 (3)	0.000 (3)	−0.003 (4)
C3	0.075 (5)	0.055 (5)	0.039 (4)	0.003 (4)	0.009 (4)	0.003 (3)
C17	0.048 (4)	0.069 (5)	0.052 (4)	0.003 (4)	−0.007 (3)	−0.001 (4)
C21	0.073 (5)	0.085 (7)	0.057 (5)	−0.006 (5)	0.015 (4)	0.015 (4)
C2	0.082 (5)	0.061 (5)	0.036 (4)	−0.007 (4)	−0.008 (3)	0.000 (4)
C20	0.051 (4)	0.072 (5)	0.045 (4)	0.002 (4)	0.012 (3)	−0.002 (4)
C23	0.090 (6)	0.065 (5)	0.045 (4)	0.011 (5)	−0.011 (4)	0.006 (4)
C22	0.087 (6)	0.139 (9)	0.071 (6)	0.021 (7)	0.037 (5)	0.009 (6)
C24	0.104 (6)	0.063 (7)	0.075 (6)	0.002 (5)	0.009 (5)	−0.021 (5)

### Geometric parameters (Å, º)

**Table:**

Br—C1	1.901 (7)	C5—C4	1.423 (9)
O1—C10	1.432 (7)	C12—H12	0.9800
O1—N2	1.475 (6)	C16—C17	1.523 (10)
N3—C13	1.369 (10)	C16—C23	1.526 (10)
N3—C24	1.453 (10)	C16—C15	1.540 (10)
N3—C14	1.465 (9)	C16—H16	0.9800
O2—C13	1.212 (9)	C15—H15A	0.9700
N2—C14	1.502 (9)	C15—H15B	0.9700
N2—C12	1.507 (8)	C7—H7	0.9300
N1—C7	1.362 (9)	C4—C3	1.401 (11)
N1—C4	1.362 (9)	C18—C17	1.526 (10)
N1—HN1	0.8929	C18—H18A	0.9700
C14—C15	1.535 (9)	C18—H18B	0.9700
C14—C19	1.576 (10)	C3—C2	1.365 (11)
C6—C1	1.381 (10)	C3—H3	0.9300
C6—C5	1.394 (10)	C17—H17A	0.9700
C6—H6	0.9300	C17—H17B	0.9700
C9—C8	1.508 (9)	C21—C20	1.537 (12)
C9—C10	1.519 (9)	C21—H21A	0.9600
C9—H9A	0.9700	C21—H21B	0.9600
C9—H9B	0.9700	C21—H21C	0.9600
C1—C2	1.413 (11)	C2—H2	0.9300
C11—C12	1.519 (9)	C20—C22	1.525 (10)
C11—C10	1.524 (9)	C20—H20	0.9800
C11—H11A	0.9700	C23—H23A	0.9600
C11—H11B	0.9700	C23—H23B	0.9600
C10—H10	0.9800	C23—H23C	0.9600
C8—C7	1.368 (9)	C22—H22A	0.9600
C8—C5	1.426 (9)	C22—H22B	0.9600
C13—C12	1.506 (11)	C22—H22C	0.9600
C19—C18	1.519 (10)	C24—H24A	0.9600
C19—C20	1.540 (10)	C24—H24B	0.9600
C19—H19	0.9800	C24—H24C	0.9600
			
C10—O1—N2	109.3 (4)	C17—C16—H16	108.6
C13—N3—C24	121.2 (7)	C23—C16—H16	108.6
C13—N3—C14	113.9 (6)	C15—C16—H16	108.6
C24—N3—C14	124.5 (7)	C14—C15—C16	114.8 (5)
O1—N2—C14	111.0 (5)	C14—C15—H15A	108.6
O1—N2—C12	104.6 (4)	C16—C15—H15A	108.6
C14—N2—C12	107.2 (5)	C14—C15—H15B	108.6
C7—N1—C4	107.9 (6)	C16—C15—H15B	108.6
C7—N1—HN1	130.2	H15A—C15—H15B	107.5
C4—N1—HN1	121.3	N1—C7—C8	111.6 (6)
N3—C14—N2	104.1 (5)	N1—C7—H7	124.2
N3—C14—C15	110.2 (5)	C8—C7—H7	124.2
N2—C14—C15	113.9 (5)	N1—C4—C3	130.3 (7)
N3—C14—C19	111.1 (5)	N1—C4—C5	108.1 (6)
N2—C14—C19	108.3 (5)	C3—C4—C5	121.6 (7)
C15—C14—C19	109.1 (5)	C19—C18—C17	112.5 (6)
C1—C6—C5	119.2 (6)	C19—C18—H18A	109.1
C1—C6—H6	120.4	C17—C18—H18A	109.1
C5—C6—H6	120.4	C19—C18—H18B	109.1
C8—C9—C10	113.7 (5)	C17—C18—H18B	109.1
C8—C9—H9A	108.8	H18A—C18—H18B	107.8
C10—C9—H9A	108.8	C2—C3—C4	118.5 (7)
C8—C9—H9B	108.8	C2—C3—H3	120.7
C10—C9—H9B	108.8	C4—C3—H3	120.7
H9A—C9—H9B	107.7	C16—C17—C18	111.2 (5)
C6—C1—C2	121.4 (7)	C16—C17—H17A	109.4
C6—C1—Br	120.7 (6)	C18—C17—H17A	109.4
C2—C1—Br	117.9 (6)	C16—C17—H17B	109.4
C12—C11—C10	101.5 (5)	C18—C17—H17B	109.4
C12—C11—H11A	111.5	H17A—C17—H17B	108.0
C10—C11—H11A	111.5	C20—C21—H21A	109.5
C12—C11—H11B	111.5	C20—C21—H21B	109.5
C10—C11—H11B	111.5	H21A—C21—H21B	109.5
H11A—C11—H11B	109.3	C20—C21—H21C	109.5
O1—C10—C9	107.0 (5)	H21A—C21—H21C	109.5
O1—C10—C11	102.8 (5)	H21B—C21—H21C	109.5
C9—C10—C11	114.9 (5)	C3—C2—C1	120.5 (7)
O1—C10—H10	110.6	C3—C2—H2	119.7
C9—C10—H10	110.6	C1—C2—H2	119.7
C11—C10—H10	110.6	C22—C20—C21	109.1 (8)
C7—C8—C5	105.6 (6)	C22—C20—C19	110.7 (7)
C7—C8—C9	126.7 (6)	C21—C20—C19	114.7 (6)
C5—C8—C9	127.7 (6)	C22—C20—H20	107.3
O2—C13—N3	125.9 (8)	C21—C20—H20	107.3
O2—C13—C12	126.4 (7)	C19—C20—H20	107.3
N3—C13—C12	107.6 (6)	C16—C23—H23A	109.5
C18—C19—C20	114.3 (6)	C16—C23—H23B	109.5
C18—C19—C14	110.7 (6)	H23A—C23—H23B	109.5
C20—C19—C14	115.3 (6)	C16—C23—H23C	109.5
C18—C19—H19	105.2	H23A—C23—H23C	109.5
C20—C19—H19	105.2	H23B—C23—H23C	109.5
C14—C19—H19	105.2	C20—C22—H22A	109.5
C6—C5—C4	118.7 (6)	C20—C22—H22B	109.5
C6—C5—C8	134.6 (6)	H22A—C22—H22B	109.5
C4—C5—C8	106.7 (6)	C20—C22—H22C	109.5
C13—C12—N2	105.9 (6)	H22A—C22—H22C	109.5
C13—C12—C11	113.3 (6)	H22B—C22—H22C	109.5
N2—C12—C11	105.8 (5)	N3—C24—H24A	109.5
C13—C12—H12	110.5	N3—C24—H24B	109.5
N2—C12—H12	110.5	H24A—C24—H24B	109.5
C11—C12—H12	110.5	N3—C24—H24C	109.5
C17—C16—C23	111.4 (6)	H24A—C24—H24C	109.5
C17—C16—C15	109.2 (6)	H24B—C24—H24C	109.5
C23—C16—C15	110.3 (6)		
			
C10—O1—N2—C12	−14.5 (6)	C8—C5—C6—C1	179.1 (7)
C10—O1—N2—C14	100.9 (5)	C4—C5—C8—C7	−0.1 (7)
N2—O1—C10—C9	155.8 (5)	C4—C5—C8—C9	178.3 (6)
N2—O1—C10—C11	34.4 (6)	C6—C5—C8—C7	179.7 (8)
C7—N1—C4—C3	−179.9 (8)	C6—C5—C8—C9	−1.9 (13)
C7—N1—C4—C5	−2.0 (8)	N1—C7—C8—C5	−1.2 (8)
C4—N1—C7—C8	2.0 (8)	N1—C7—C8—C9	−179.6 (6)
O1—N2—C12—C11	−11.6 (7)	C5—C8—C9—C10	−78.1 (9)
O1—N2—C12—C13	109.0 (5)	C7—C8—C9—C10	100.1 (8)
C14—N2—C12—C11	−129.6 (6)	C8—C9—C10—O1	74.7 (7)
C14—N2—C12—C13	−9.0 (6)	C8—C9—C10—C11	−171.8 (6)
O1—N2—C14—N3	−103.0 (5)	O1—C10—C11—C12	−39.7 (7)
O1—N2—C14—C15	17.2 (7)	C9—C10—C11—C12	−155.6 (6)
O1—N2—C14—C19	138.7 (5)	C10—C11—C12—N2	31.3 (7)
C12—N2—C14—N3	10.8 (6)	C10—C11—C12—C13	−84.4 (7)
C12—N2—C14—C15	130.9 (6)	N2—C12—C13—O2	−175.3 (7)
C12—N2—C14—C19	−107.6 (5)	N2—C12—C13—N3	3.5 (7)
C14—N3—C13—O2	−177.5 (7)	C11—C12—C13—O2	−59.8 (10)
C14—N3—C13—C12	3.6 (8)	C11—C12—C13—N3	119.1 (6)
C24—N3—C13—O2	−4.7 (12)	N2—C14—C15—C16	67.4 (8)
C24—N3—C13—C12	176.5 (6)	N3—C14—C15—C16	−176.0 (6)
C13—N3—C14—N2	−9.2 (7)	C19—C14—C15—C16	−53.7 (7)
C13—N3—C14—C15	−131.7 (6)	N2—C14—C19—C18	−71.8 (7)
C13—N3—C14—C19	107.2 (7)	N2—C14—C19—C20	59.8 (7)
C24—N3—C14—N2	178.3 (6)	N3—C14—C19—C18	174.4 (6)
C24—N3—C14—C15	55.8 (9)	N3—C14—C19—C20	−54.0 (8)
C24—N3—C14—C19	−65.3 (8)	C15—C14—C19—C18	52.6 (7)
Br—C1—C2—C3	177.9 (6)	C15—C14—C19—C20	−175.8 (6)
C6—C1—C2—C3	−2.2 (12)	C14—C15—C16—C17	55.4 (8)
Br—C1—C6—C5	−177.7 (5)	C14—C15—C16—C23	178.1 (6)
C2—C1—C6—C5	2.4 (11)	C15—C16—C17—C18	−55.5 (8)
C1—C2—C3—C4	0.6 (12)	C23—C16—C17—C18	−177.4 (6)
C2—C3—C4—N1	178.3 (7)	C16—C17—C18—C19	58.6 (8)
C2—C3—C4—C5	0.7 (11)	C17—C18—C19—C14	−56.6 (8)
N1—C4—C5—C6	−178.5 (6)	C17—C18—C19—C20	171.3 (6)
N1—C4—C5—C8	1.3 (8)	C14—C19—C20—C21	−89.9 (8)
C3—C4—C5—C6	−0.4 (10)	C14—C19—C20—C22	146.0 (7)
C3—C4—C5—C8	179.4 (7)	C18—C19—C20—C21	40.0 (9)
C4—C5—C6—C1	−1.1 (10)	C18—C19—C20—C22	−84.1 (9)

### Hydrogen-bond geometry (Å, º)

Cg3 is the centroid of the N2/C12/C13/N3/C14 five-membered imidazolidinone ring.

**Table:**

*D*—H···*A*	*D*—H	H···*A*	*D*···*A*	*D*—H···*A*
N1—H*N*1···N2^i^	0.89	2.34	3.087 (8)	141
C3—H3···O2^ii^	0.93	2.42	3.292 (9)	156
C16—H16···O1	0.98	2.55	3.091 (8)	115
C20—H20···N3	0.98	2.54	3.032 (10)	111
C21—H21*A*···N2	0.96	2.60	3.236 (9)	124
N1—H*N*1···O1^i^	0.89	2.66	3.053 (8)	108
C20—H20···*Cg*3	0.98	2.41	2.866 (8)	104

Symmetry codes: (i) −*x*+1, *y*+1/2, −*z*+1; (ii) *x*, *y*, *z*−1.
